# An App for Navigating Patient Transportation and Acute Stroke Care in Northwestern Ontario Using Machine Learning: Retrospective Study

**DOI:** 10.2196/54009

**Published:** 2024-08-01

**Authors:** Ayman Hassan, Rachid Benlamri, Trina Diner, Keli Cristofaro, Lucas Dillistone, Hajar Khallouki, Mahvareh Ahghari, Shalyn Littlefield, Rabail Siddiqui, Russell MacDonald, David W Savage

**Affiliations:** 1 Thunder Bay Regional Health Sciences Centre Thunder Bay, ON Canada; 2 Thunder Bay Regional Health Research Institute Thunder Bay, ON Canada; 3 Northern Ontario School of Medicine University Thunder Bay, ON Canada; 4 University of Doha for Science and Technology Doha Qatar; 5 Lakehead University Thunder Bay, ON Canada; 6 Ornge Mississauga, ON Canada

**Keywords:** stroke care, acute stroke, northwestern, Ontario, prediction, models, machine learning, stroke, cardiovascular, brain, neuroscience, TIA, transient ischemic attack, coordinated care, navigation, navigating, mHealth, mobile health, app, apps, applications, geomapping, geography, geographical, location, spatial, predict, predictions, predictive

## Abstract

**Background:**

A coordinated care system helps provide timely access to treatment for suspected acute stroke. In Northwestern Ontario (NWO), Canada, communities are widespread with several hospitals offering various diagnostic equipment and services. Thus, resources are limited, and health care providers must often transfer patients with stroke to different hospital locations to ensure the most appropriate care access within recommended time frames. However, health care providers frequently situated temporarily (locum) in NWO or providing care remotely from other areas of Ontario may lack sufficient information and experience in the region to access care for a patient with a time-sensitive condition. Suboptimal decision-making may lead to multiple transfers before definitive stroke care is obtained, resulting in poor outcomes and additional health care system costs.

**Objective:**

We aimed to develop a tool to inform and assist NWO health care providers in determining the best transfer options for patients with stroke to provide the most efficient care access. We aimed to develop an app using a comprehensive geomapping navigation and estimation system based on machine learning algorithms. This app uses key stroke-related timelines including the last time the patient was known to be well, patient location, treatment options, and imaging availability at different health care facilities.

**Methods:**

Using historical data (2008-2020), an accurate prediction model using machine learning methods was developed and incorporated into a mobile app. These data contained parameters regarding air (Ornge) and land medical transport (3 services), which were preprocessed and cleaned. For cases in which Ornge air services and land ambulance medical transport were both involved in a patient transport process, data were merged and time intervals of the transport journey were determined. The data were distributed for training (35%), testing (35%), and validation (30%) of the prediction model.

**Results:**

In total, 70,623 records were collected in the data set from Ornge and land medical transport services to develop a prediction model. Various learning models were analyzed; all learning models perform better than the simple average of all points in predicting output variables. The decision tree model provided more accurate results than the other models. The decision tree model performed remarkably well, with the values from testing, validation, and the model within a close range. This model was used to develop the “NWO Navigate Stroke” system. The system provides accurate results and demonstrates that a mobile app can be a significant tool for health care providers navigating stroke care in NWO, potentially impacting patient care and outcomes.

**Conclusions:**

The NWO Navigate Stroke system uses a data-driven, reliable, accurate prediction model while considering all variations and is simultaneously linked to all required acute stroke management pathways and tools. It was tested using historical data, and the next step will to involve usability testing with end users.

## Introduction

In Canada, annual stroke occurrence rates have increased from one stroke occurrence every 9 minutes to every 5 minutes in less than a decade [1,2]. In the province of Ontario, stroke is the third leading cause of death and the leading cause of adult disability [3]. There are an estimated 25,500 new stroke events and 15,500 hospital inpatient admissions in Ontario each year [4].

A coordinated system of care is essential for providing timely access to treatment for patients who present with a suspected acute stroke, transient ischemic attack, or minor nondisabling stroke. It is estimated that for every minute of delay in treating an acute ischemic stroke, 1.9 million brain cells die, 13.8 billion synapses are damaged, and 12 kilometers of axonal fibers are lost [1]. Patients eligible for time-sensitive therapies should be transported to the closest hospital capable of providing services for the diagnosis and treatment of stroke [5].

**Figure 1:**
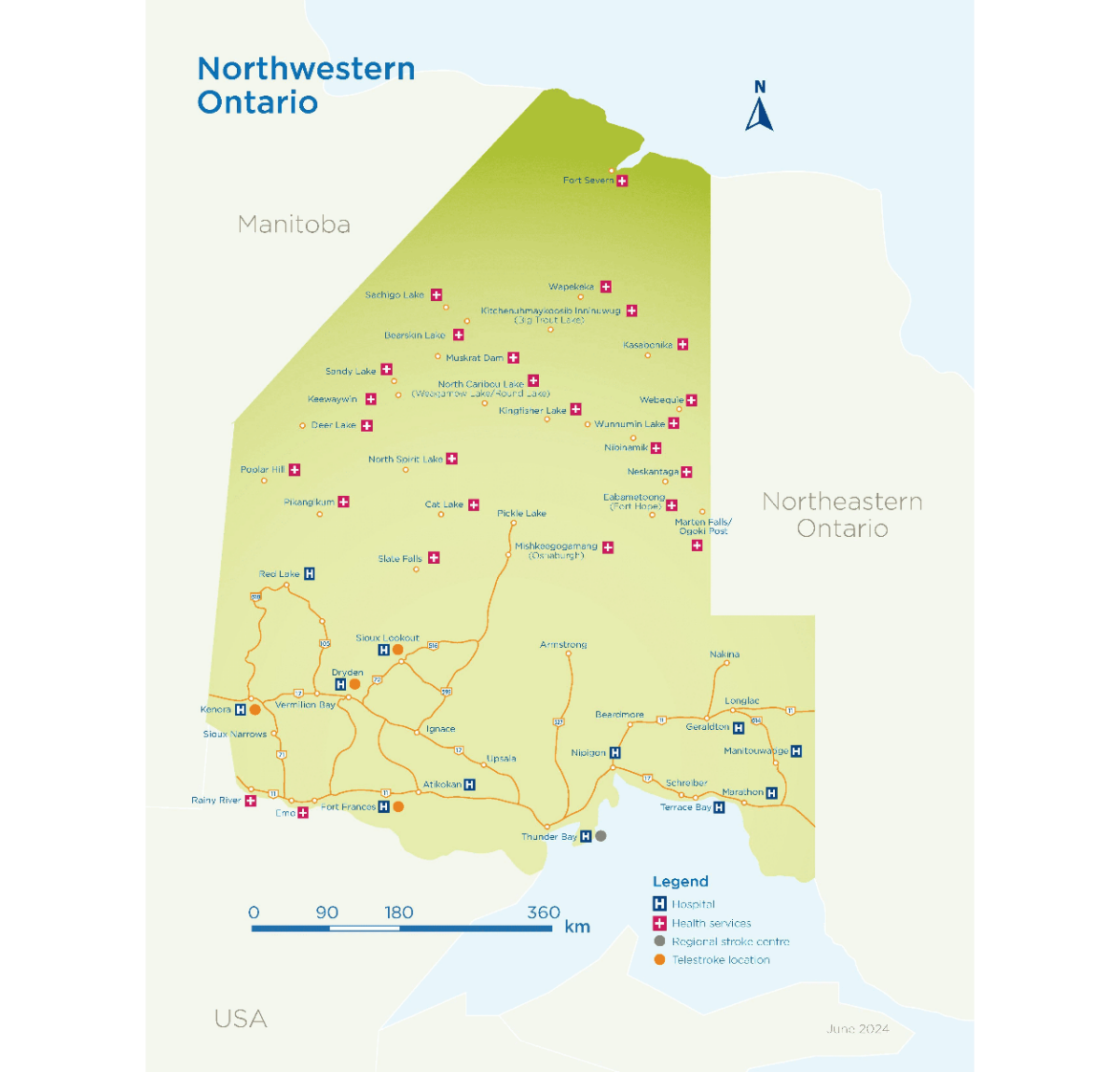
Map of Northwestern Ontario, Canada, showing the hospital or acute health services that have essential acute stroke protocols implemented among the various communities.

Patients accessing care for stroke in Northwestern Ontario (NWO), Canada, have particular challenges. NWO is a vast geographic area, covering approximately 458,010 km^2^ in the northwest region of the province (Figure 1) [6]. The health status of NWO residents differs from that of the remaining population in Ontario and Canada. Health indicators typically show worse outcomes for NWO residents when compared with provincial and national indicators. NWO residents have a lower life expectancy, a higher mortality rate, and are more likely to die as a result of circulatory disease [7]. Furthermore, indigenous peoples are more likely to have chronic diseases than nonindigenous peoples [7]. The Heart and Stroke Foundation of Canada reports that indigenous peoples have a 50% higher heart disease rate and are twice as likely to die from a stroke as nonindigenous peoples [8]. Statistics on the health of Indigenous peoples in NWO, who represent 18.3% (2.4% provincially) of the region’s population are significantly underreported [7]. Therefore, due to the region’s geography and the relative health status of its residents, it is important to ensure timely access to acute stroke care for this population.

Given that many NWO communities are only accessible by air, Ontario’s air ambulance program (Ornge) previously compared estimated and actual patient transfer times for sending-to-receiving hospital pairs. This prior work and resulting analyses revealed that estimates of transport time were inconsistent and not always comparable to actual times [9,10]. This work also revealed that the flight planner’s estimation of “time to definitive care” was based on experience, subjective judgments, and consultations with pilots and transport medicine physicians, which led to imprecision in time estimates used to make vehicle allocation decisions. Ornge has since developed a decision support tool that uses historical dispatch and call data to generate relevant time estimates for transfer between hospital pairs. However, this tool primarily focuses on generating time estimations for a given sending and receiving hospital pair and does not consider hospital capabilities and services.

## Methods

### Ethical Considerations

Ethics approval for this study was obtained from the Lakehead University Research Ethics Board (approval number 1467257) which functions in accordance with the principles of the Declaration of Helsinki and the Tri-Council Policy Statement: Ethical Conduct for Research Involving Humans 2. As this project involved retrospective and deidentified data collection with no ability to reidentify patients, no impact on their medical rights, or pose additional risks, the Lakehead University Research Ethics Board approved a waiver for obtaining informed consent from participants. The data collected from databases at Ornge and land ambulance services were all deidentified.

### Overview of Data Analysis

**Figure 2:**
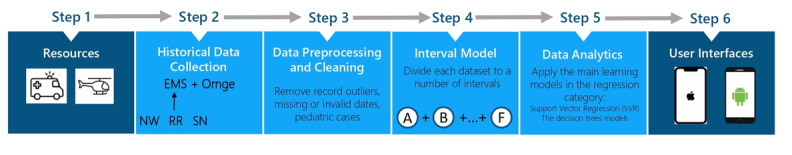
The overall architecture of the data analytics, starting with the collection of transportation timelines for air and land ambulances and concluding with the development of the Northwestern Ontario Navigate app to assist health care providers treating patients with stroke with the most efficient patient transfer options. EMS: emergency medical service; NW: Northwest; RR: Rainy River; SN: Superior North.

Figure 2 presents a schematic of the data analysis process used in this study.

### Data Set

The data set used in this study was obtained from air medical transport (Ornge) and land medical transport (3 services) data collected over 2008-2020 (Textbox 1). The land medical transport data set contains 3000 samples, and each sample has 5 input variables. The Ornge data set contains 15,400 samples, with 12 input variables. Both the Ornge and land medical transport data sets were distributed as follows: training (35%), testing (35%), and validation (30%).

The terms “accurate,” “reliable,” and “validation” are used from a system engineering point of view. Accurate refers to the degree of closeness between a measurement (eg, the predicted value) and its true value (eg, the historical data). Reliable refers to the consistency and performance of the model, or the percentage of nonfailures that occur within a unit of time. Validation is the process of retesting a machine learning system to validate the quality of the system using different data and parameters than that used in the testing stage, thus ensuring trained models are built with high quality, compliant, and accepted by end users to increase their adoption.

**List of data points collected from the historical data set obtained from land transport services that were used to train, test, and validate the machine learning model used for the Northwestern Ontario Navigate Stroke app.**

**Land medical transport data set (3 services)**

- Sending hospital
- Receiving facility or destination
- Call received date or time
- Arrive destination date or time
- Depart destination date or time

**Ornge medical transport data set**

- Sending facility
- Receiving facility
- Call accepted date or time
- Crew notified
- Depart base date or time
- Arrive pickup landing date or time
- Arrive patient site date or time
- Depart patient site date or time
- Depart landing site date or time
- Arrive destination landing site date or time
- Arrive receiving site date or time
- Transfer of care date or time

### Data Preprocessing and Cleaning

The preprocessing phase consisted of initial data cleaning to remove redundant, missing, or erroneous data that might have been generated during the data collection process. The data set consists of deidentified data from January 1, 2008, to December 30, 2020, including the pickup location of the patient, the destination of the patient (either a hospital location, airport, or helipad), and the dispatch priority. The data set was cleaned to include only hospital locations that would have a recommended “driving” journey to definitive stroke care based on NWO limitations.

### Data Fusion and Time-Interval Organization

**Figure 3:**
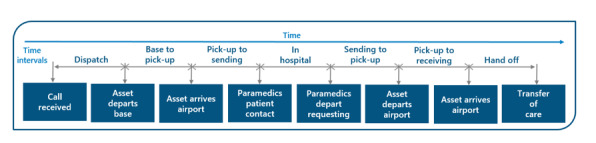
Time intervals used to divide the “total time to definitive care” per patient transport process, starting from the time the call is received to the time the patient is transferred to care. This approach was used to overcome the lack of data that occurred once the patient journeys involving both air and land were merged.

Data were merged when both Ornge air services and land ambulance medical transport were involved in patient transport. Upon merging the data sets, the overall data set was significantly reduced, and there was a clear lack of significant data that could be used for developing average times for all patient journeys. Therefore, a time-interval approach was used from call time to transfer of care, as previously used by Giang et al [10]. This approach is displayed schematically in Figure 3.

### Data Analytics and Comparison With Prior Work

Different models, such as basic, statistical, machine learning (support vector regression [SVR], random forest, gradient boosting, etc), and deep learning (long short-term memory and gated recurrent unit) models, were compared to identify the best opportunity for obtaining high-precision predictions.

There are different learning models for regression and classification. Given the nature of the data sets and the continuous nature of the variables to be predicted, a regression learning model was used. The regression model was chosen by considering the complexity of each model option, as well as the elements and values of the data to be analyzed. The regression model was trained based on a history of observations to produce a predictive model capable of explaining a continuous output variable, for example, a variable defined in a set of real numbers. Among the various learning models available, this study used two main regression learning models: SVR and decision tree models.

Data that were used in the learning process were also chosen randomly (unordered) from the data set. Quantitative forecasting methods were applied, and only the learning models retained in the adjustment step (random forest, multiple linear regression, and SVR with a radial basis function kernel) were tested.

The random forest method is an ensemble learning method used for both classification and regression tasks [11]. Linear regression is a type of general linear model that is often used to predict one variable from another. SVR is similar to linear regression; however, unlike other regression models that aim to minimize the error between the real and predicted values, SVR focuses on fitting a hyperplane (straight line) that best represents the relationship between the input variables and the target variable. The radial basis function kernel is a key component of SVR that enables the algorithm to capture nonlinear relationships between variables.

The output or predicted parameter of the algorithms is the time of transfer of the patient from the sending to receiving facilities via either land or air transportation. Python and Anaconda were used at Lakehead University for data analysis and development of the machine learning model.

## Results

### Data Collection

The prediction model is based on approximately 13 years of historical data from land and air medical transport services (Table 1) serving NWO. The organizations that contributed to the data set included Ornge, Northwest Emergency Medical Service (EMS), Superior North EMS, and Rainy River District EMS. Ornge serves the region with land ambulance services from Northwest, Superior North, and Rainy River EMS providers.

**Table 1:** Breakdown of the historical data sets, sources, timestamps, and number of records that were used to develop the machine learning model for Northwestern Ontario Navigate Stroke.

Data set	Timestamp	Number of records (N=70,623)
Ornge	January 1, 2008-December 31, 2018	67,412 (1815 stroke or TIA^a^)
NW^b^ EMS^c^	November 9, 2009-October 26, 2019	926
SN^d^ EMS	October 1, 2012-September 1, 2019	2014
RR^e^ District EMS	January 8, 2015-February 24, 2020	271

^a^TIA: transient ischemic attack.

^b^NW: Northwest.

^c^EMS: emergency medical service.

^d^SN: Superior North.

^e^RR: Rainy River.

### Analysis Results

**Figure 4:**
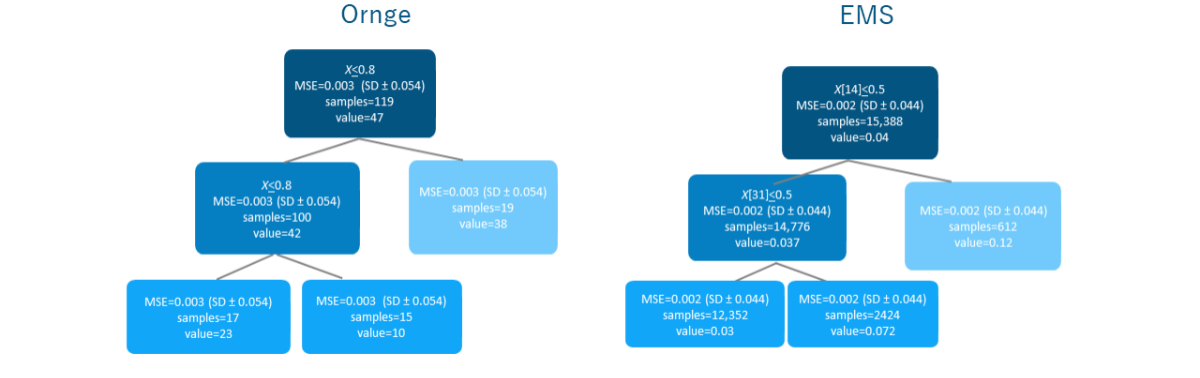
Decision tree model using the K1 MSE for EMS and Ornge data, illustrating the decision tree learning model where X indicates the variables used to make decisions at each node of the tree, samples are the number of the data set in cases represented by the node, and values are those associated with target variables. EMS: emergency medical service; MSE: mean square error.

When considering the statistical analysis approach (machine learning method or simple statistics method), the machine learning approach is opted for. This approach met the needs of the project (machine learning requires updated data to develop a prediction system, not a manual update and revalidation as simple statistics would). Tables 2 and 3 show the analysis results for each characteristic depending on the transformation applied to the data (raw, normalization, and standardization). All learning models perform better than the simple average of all points in terms of predicting the output variables. Analysis of the K1 value related to the decision tree model is shown in Figure 4.

**Table 2:** Analysis of medical transport data in the prediction models.

Prediction model			Mean square error, mean (SD)
SVR^a^			0.007 (0.083)
Random forest			0.22 (0.469)
**Decision tree**			
	K1	0.002 (0.044)	
	K2	0.001 (0.032)	
	K3	0.001 (0.032)	

^a^SVR: support vector regression.

**Table 3:** Analysis of Ornge data in the prediction models.

Prediction model			Mean square error, mean (SD)
SVR^a^			0.01 (0.01)
Random forest			0.20 (0.44)
**Decision tree**			
	K1	0.003 (0.054)	
	K2	0.002 (0.044)	
	K3	0.005 (0.07)	

^a^SVR: support vector regression.

Tables 2 and 3 also present the mean square errors (MSEs) of the SVR, random forest, and decision tree models applied to the data sets. Based on the results, it was concluded that the decision tree model provides more accurate results in comparison to the other models. The decision tree model was trained and evaluated 3 times with different parameters, resulting in different MSE values. To differentiate between these values and show how the model performs when using different parameters, labels, such as K1, K2, and K3, are assigned to each MSE value.

Table 2 presents the analysis results for medical transport data in the prediction models, and Table 3 represents the analysis results for Ornge data in the prediction models. The MSE serves as a machine learning indicator that assesses the accuracy and performance of predictive models, providing a measure of the average squared difference between the actual and predicted values.

The MSEs for the SVR and random forest models in Table 2 (0.007 and 0.22, respectively) indicate high prediction errors compared with the decision tree model. The same trend is shown in Table 3, with the decision tree model achieving higher accuracy than the other models.

We note that the system has continuous learning capabilities and can learn from newly generated end user data if such data are dynamically added to the historical data.

### Testing, Validation, and the Prediction Model

The results of the testing-validation-model process show that the decision tree model performed remarkably well. The values from testing, validation, and the model all resulted within a close range. Table 4 demonstrates how the trained model is applied to new data sets to assess its performance and effectiveness in making predictions using a sample of data from the land EMS data set. The proposed validated prediction model was used at the back end of the developed system to predict the time of transfer for patients from sending to receiving facilities using either land or air transportation for the different scenarios encountered by end users. The results show that the system can reliably predict the transfer time based on the 13-year historical data set with good accuracy, as demonstrated by the sample data in Table 4.

**Table 4:** Sample data demonstrating the test-validation-prediction model process for land emergency medical service.

Sending facility and respective receiving facility			Testing^a^		Validation^a^		Prediction model^a^
**Atikokan General Hospital**							
	La Verendrye General Hospital	102		102		102	
	Thunder Bay Regional	112.9		130		115.9	
**Dryden Regional Health Center**							
	Lake of the Woods	116.9		123.9		115.3	
	La Verendrye General Hospital	177		177		177	
	Sioux Lookout	94.2		83.1		93.2	
**Lake of the Woods**							
	Dryden Regional Health Center	111.2		111.2		111.2	
	Lake of the Woods	45.3		45.3		45.3	
**Rainy River Health Center**							
	Dryden Regional Health Center	106		106		106	
	La Verendrye General Hospital	39.9		42.1		39.9	
	Lake of the Woods	191.4		200.7		191.3	
**Red Lake Hospital**							
	Dryden Regional Health Center	56.2		56.2		56.2	
**Sioux Lookout**							
	Dryden Regional Health Center	98.8		98.8		98.8	

^a^All measurements are in minutes.

### System Development

The proposed system uses a mobile app and a web-based application. The Ionic framework was used for cross-platform development on both Android and iOS devices. Starting as a simple one-screen app with basic mapping capabilities, the app grew to be a complex system, offering different experiences based on stroke symptoms when the patient was last known to be well, and the patient’s location.

NWO Navigate Stroke uses a database to store and retrieve information about stroke services and historical emergency services data. The data are stored in Firebase’s Cloud Firestore. Information on services, locations, and historical data are stored locally on the device, with live data pulls for additions or modifications. Storing data locally allows for shorter computational times and lower database costs. To assess use, information logs, including when the system was used, which pages were accessed, start and end locations, time spent on the app, and device specifications, are recorded. Each time the app is closed, records are automatically uploaded to the database.

In the infrequent instances (less than 10%) when no historical data are available (eg, new health care facility in the region, new transportation protocol approved or developed), the Google Maps application programming interface (API) is used to estimate travel times. The Google Maps API allows a digital map to be displayed, with custom markers for gathering travel information. By using the digital map, end users can explore resources available in the region and view possible routes after a destination has been selected. The Google Maps API also provides the distance by road and the estimated trip duration, which is used to estimate ambulance travel times. Weather information, such as the current temperature and current conditions, is obtained through the OpenWeatherMap API and used to display weather at the patient’s location to indicate weather variability that could cause transportation complications.

## Discussion

### Principal Findings

In developing the NWO Navigate Stroke system, understanding different capabilities at hospital locations related to stroke care (eg, imaging capabilities) was essential. While the research project started as a geomapping and estimation system, it was realized that tools, algorithms, and agreements needed to be embedded. These additions were important for end users with a low incidence of patients with stroke across a complex medical transport ecosystem. For each hospital location, various stroke-specific processes and protocols, route options, and communication steps to access stroke care had to be understood and coded. With this information, various time frames for stroke treatment (0-6 h, 6-24 h, >24 h, 0-48 h, >48 h) were associated with hospitals and health service locations. The system was adapted to offer multiple options depending on the patient’s location. Although the system provides amalgamated resources for acute stroke management, it purposefully does not provide clinical decisions for the user.

The system works as designed, to aid in transportation transparency. User inputs of clinical assessment outcomes (eg, when the patient was last known to be well, the outcome of stroke assessment, and stroke severity assessment for large vessel occlusion) are used to determine and display the next steps. Clinical support includes tips in determining stroke symptom onset and direct links to Canadian Stroke Best Practice Recommendations: Acute Stroke [7]. The system is unique, as it addresses navigation and communication, alongside resources and possible time frames for treatment. The system is intended to give immediate access to information on services for accessing stroke care.

**Figure 5:**
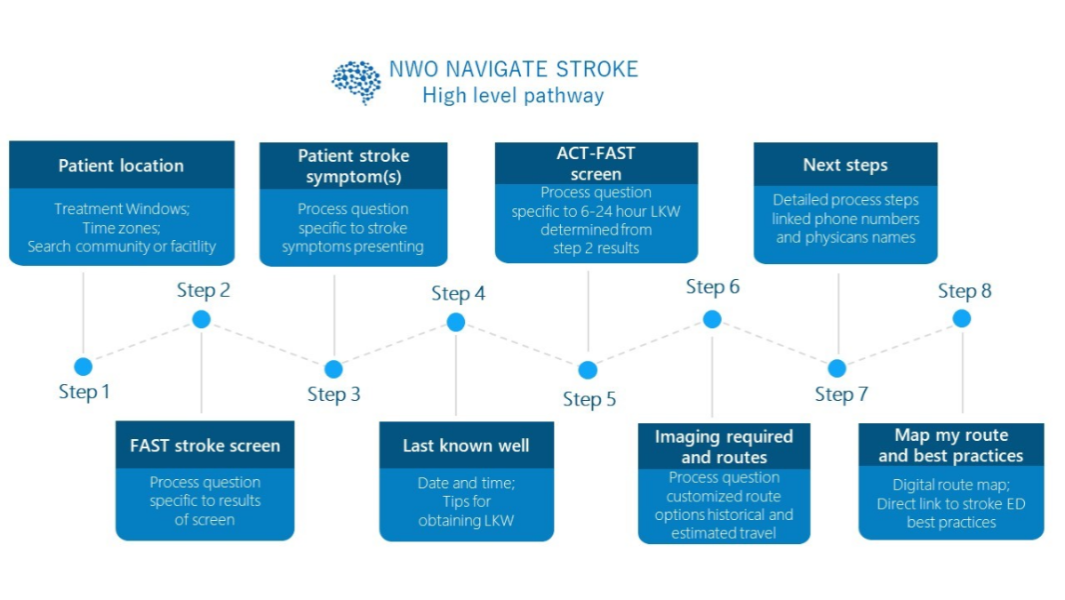
Overview of the algorithm of the NWO Navigate Stroke app for guiding the health care provider in treating a patient with stroke with the most efficient patient transfer option, with a consideration of key factors such as patient location, last time the patient was known to be well, need for imaging, and stroke symptoms. LKW: last known well; NWO: Northwestern Ontario.

**Figure 6:**
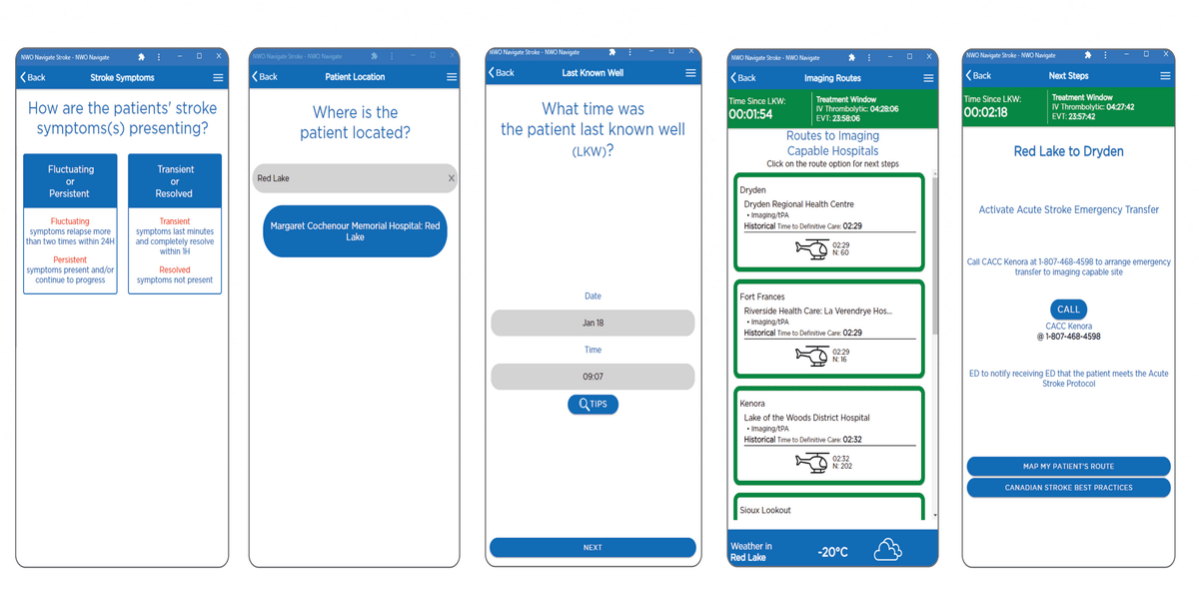
Screenshots of the NWO Navigate Stroke app displaying an example of a patient requiring acute stroke care and transportation to another facility for treatment. The pathway shows that the patient’s symptoms are assessed, their location is entered, and their LKW time is inputted; this leads to the calculation of possible routes the patient may be transferred, along with the method (air or land) and expected timeline to care. Once a route has been selected, the final screen will prompt the end user to activate the transfer process. LKW: last known well; NWO: Northwestern Ontario.

The system’s pathway has 8 steps to ultimately provide guidance on the best route for patients with stroke care (Figure 5). To find a transportation route, the end user inputs the patient’s current location and assessment results (involving a stroke screening tool, presenting stroke symptoms, and when the patient was last known to be well). The time when the patient was last known to be well determines the time remaining for treatment; a visual countdown clock of time remaining for each treatment option (thrombolytics and endovascular therapy) is shown based on the information entered, which remains on the screen throughout the entry process. If the time the patient was last known to be well is 6-24 hours, the user is asked for the results of the large vessel occlusion screening tool to determine the patient’s pathway. The end user is guided through applicable screens involving imaging questions, process steps, and options for transportation. Route options, process references, treatment options, travel times, and a list of capable hospital locations are provided. Once the user has an estimation of transportation time, a judgment can be made against the treatment windows and the best transportation location (telestroke hospital or regional stroke center). A menu provides an opportunity to explore the digital stroke services map, stroke icon legends, Canadian Stroke Best Practices guidance, an application tutorial, frequently asked questions, and contact information; Figure 6 contains screenshots of the app with an example of a patient.

### Future Directions and Limitations

The NWO Navigate Stroke system has been developed to provide efficient transportation assistance for stroke care only in NWO but has the potential for expansion to include other acute services that could benefit from efficient transportation assistance such as cardiac or trauma services. At this stage, the system does not include health care facilities that are not available within the Canadian health care system context (eg, facilities in the United States), even though these facilities may be geographically closer than other locations in the system. However, it should be noted that the system can be further developed to include such facilities and other acute services. Although no patient data were built in, future expansion of the app could include patient data, live data feeds, or other features as identified by end users over time. Such additions would need to consider federal and provincial data privacy legislation and encryption and security needs.

### Contributions and Strengths

The NWO Navigate Stroke system unites prior research conducted by Ornge with 3 land-only medical transport systems operating within NWO and Google Map estimates to provide a consolidated view of transport options. Prior work conducted by Ornge focused on using historical data of their land and air ambulance system to understand interfacility patient transport processes [9] but did not include other data sources, such as other prehospital providers, which can be crucial when considering efficient patient care. The developed system has many strengths, including the following: (1) Integration of interregional and interprovincial transportation pathways, making this knowledge easily accessible for the user. (2) Inclusion of acute stroke assessment tools, such as FAST (symptom checklist for stroke: Facial drooping, Arm weakness, Speech difficulties, and Time) and ACT-FAST (Ambulance Clinical Triage for Acute Stroke Treatment) for large vessel occlusion screening used in anterior circulation strokes, which are an integral part of the proposed system and can aid in the learning process for small-volume health facilities [12,13]. (3) Links to the Canadian Heart and Stroke’s Stroke Best Practice Recommendations including Emergency Services Medical Management [12]. (4) Ability to contact the consulting physician for transport acceptance or consultation. (5) Provision of pathways for differentiating symptoms that include suspected or diagnosed acute stroke or subacute stroke. (6) An algorithm to predict pathways based on computerized tomography availability. (7) An adaptive machine learning algorithm that can continuously learn from data generated by the end user, which is dynamically added to historical data to enhance system precision. (8) Predictions based on when the patient was last known to be well, with color-coded windows for acute management based on the time of onset.

### Conclusion

By using data from medical transportation systems in NWO, a system was developed that can be used by health care providers when deciding on optimal transfer for patients experiencing a suspected acute stroke, with all required tools being simply a touch away. Usability testing is needed to ensure that the system meets the needs of end users. Feedback will be obtained through multiple methods during the release of the app, including a live educational tour of hospitals (ongoing since summer 2022). Following the release of the app, quality improvements will be made based on feedback, with updates to pathways based on best practices.

## Abbreviations

ACT-FAST: Ambulance Clinical Triage for Acute Stroke Treatment
API: application programming interface
EMS: emergency medical service
MSE: mean square error
NWO: Northwestern Ontario
SVR: support vector regression

## Data Availability

The data sets generated and analyzed during this study are not publicly available as they are held securely by Ornge but are available from the corresponding author on reasonable request.
